# How to compare instrumental variable and conventional regression analyses using negative controls and bias plots

**DOI:** 10.1093/ije/dyx014

**Published:** 2017-04-07

**Authors:** Neil M Davies, Kyla H Thomas, Amy E Taylor, Gemma MJ Taylor, Richard M Martin, Marcus R Munafò, Frank Windmeijer

**Affiliations:** dyx014-1 Medical Research Council Integrative Epidemiology Unit,; dyx014-2 School of Social and Community Medicine,; dyx014-3 UK Centre for Tobacco and Alcohol Studies and; dyx014-4 Department of Economics, University of Bristol, Bristol, UK

**Keywords:** Instrumental variables, negative controls, pharmacoepidemiology, causal inference

## Abstract

There is increasing interest in the use of instrumental variable analysis to overcome unmeasured confounding in observational pharmacoepidemiological studies. This is partly because instrumental variable analyses are potentially less biased than conventional regression analyses. However, instrumental variable analyses are less precise, and regulators and clinicians find it difficult to interpret conflicting evidence from instrumental variable compared with conventional regression analyses. In this paper, we describe three techniques to assess which approach (instrumental variable versus conventional regression analyses) is least biased. These techniques are negative control outcomes, negative control populations and tests of covariate balance. We illustrate these methods using an analysis of the effects of smoking cessation therapies (varenicline) prescribed in primary care.

**Key Messages**

- Clinicians and regulators struggle to interpret conflicting evidence from instrumental variable compared with conventional regression analysis.
- The relative bias of these methods can be assessed using negative control outcomes, negative control populations and tests of covariate balance.
- Researchers could report bias component plots with confidence intervals to robustly assess the relative bias due to each covariate.

## Introduction

Unmeasured or residual confounders can bias the results from observational studies of routinely collected data. For example, in pharmacoepidemiological studies, treatment choice is influenced by a number of factors (e.g. comorbidities, socioeconomic position, education) that relate to outcomes, but are often not perfectly recorded or measurable in the sorts of electronic medical records data that are used in such analyses. This ‘confounding by indication’ means that the observed association of treatment with an outcome is often an unreliable indicator of any causal adverse or beneficial effects of the treatment of interest.

**Figure 1:**
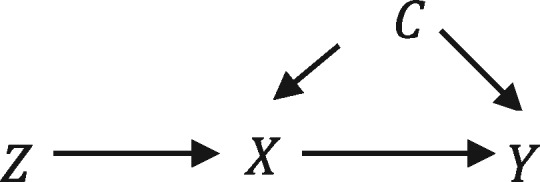
Directed acyclic graph of outcome Y, prescription X, the instrumental variable Z and a potentially unmeasured confounder C (left). Each variable’s directed effects (edges) are denoted by arrows.

This problem of ‘confounding by indication’ is illustrated in Figure 1, where the outcome *Y* is caused by the exposure *X* and the unobserved or residual confounder *C.* The association of the exposure with the outcome will be biased because they are both caused by a confounding factor *C.* Confounding by indication affects the likelihood of receiving the prescription and having the outcome, independently of the true causal effects of the prescription. Therefore using methods which adjust for confounding, such as multivariable adjusted regression or propensity score regression, when the confounding factors are either not measured or not measured sufficiently precisely can give biased estimates.^1^

Instrumental variable analysis is a statistical approach that can theoretically overcome these problems.^2–7^ Instrumental variables are defined by three assumptions: A) they are associated with the exposure of interest; B) they are not associated with confounding factors; and C) they have no direct effect on the outcome of interest.^8^^,^^9^ These assumptions are illustrated in Figure 1, where the instrument variable *Z* only affects the prescription *X.*

We can obtain a valid estimate of the effects of the exposure on the outcome using the so-called Wald estimator which identifies the effects of treatment on the risk difference scale. Denote the sub-sample averages of *Y* and *X* by ${\overset{¯}{y}}_{1}$ and ${\overset{¯}{x}}_{1}$ when Z=1 and by ${\overset{¯}{y}}_{0}$ and ${\overset{¯}{x}}_{0}$ when Z=0. The Wald estimator is then given by:

$$\hat{\psi }=\frac{{\overset{¯}{y}}_{1}-{\overset{¯}{y}}_{0}}{{\overset{¯}{x}}_{1}-{\overset{¯}{x}}_{0}}\;$$

and is consistent for the estimand:

$$\psi =\frac{E\left[Y|Z=1\right]-E\left[Y|Z=0\right]}{E\left[X|Z=1\right]-E\left[X|Z=0\right]}$$

**Figure 2:**
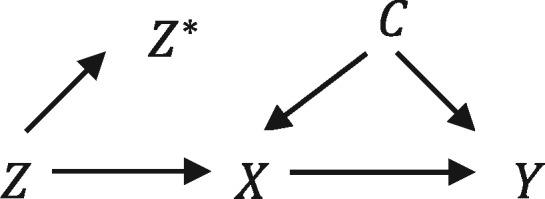
Directed acyclic graph of an analysis using the physicians’ prescriptions to their previous patients, Z* as a proxy for their preferences, the true underlying instrument, Z, which is a latent variable. The exposure, outcome and confounder are indicated as X, Y and C, respectively.

In a pharmacoepidemiological study of the effects of prescribed drugs, physicians’ preferences for particular drugs are potential instruments for the prescriptions they issue to their patients.^7^ This is because physicians’ preferences for medications affect the drugs they issue (assumption i), but the preferences themselves will not necessarily be related to their patients’ pre-existing comorbidities (assumption ii) and will not necessarily directly affect their patients’ outcomes (assumption iii). Patients generally register with their GP long before they are prescribed treatments, so their choice of GP is unlikely to be related to their GP’s preference for a specific medication, thus ensuring that using prescribing preference as an instrument for treatment received does not violate assumptions i and ii. We cannot directly measure physicians’ preferences from prescribing databases, so preferences are ‘latent variables’ indicated by *Z* in Figure 2. In the analysis of the effects of smoking cessation therapies in primary care described here, we use the physicians’ prescriptions of varenicline or nicotine replacement products to their previous patients as proxies for their preferences. Recent studies have found that physicians’ prescribing preferences could potentially be a valid instrument for prescribing of non-steroidal anti-inflammatory drugs (NSAIDs), antidepressants, smoking cessation medication and anti-psychotics.^7^^,^^10–20^ However, a study using data from German health insurance records found that physicians’ preferences are not always valid for NSAIDs.^21^ Therefore the validity of physicians’ prescribing preferences as instruments is context-dependent and needs to be assessed in new applications or data sources.

As the use of instrumental variable methods is relatively novel in epidemiology, we currently do not have sufficient information to advise policy makers and regulators about the specific situations where instrumental variable analysis is likely to provide a less biased estimate of the causal effect of a drug than conventional regression analyses. Here we describe how negative control outcomes, negative control populations and bias component plots can be used to assess the validity of instrumental variables for dealing with confounding by indication.^22^

## Methods

In this section, we describe three approaches to assess the relative bias of conventional and instrumental variable estimates by using: i) negative control outcomes; ii) negative control populations; and iii) bias component plots.

### Negative control outcomes

**Figure 3:**
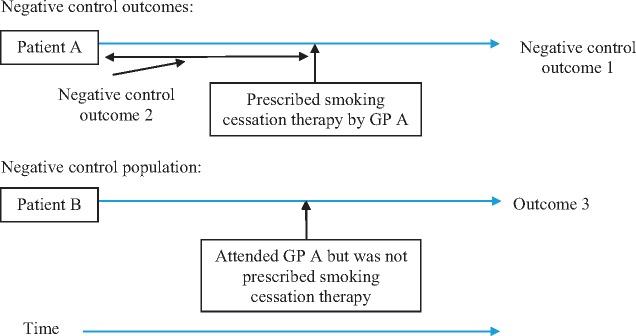
Proposed negative control outcomes and negative control populations.

One way to evaluate whether the instrumental variable assumptions hold is to investigate whether the instrumental variables are associated with negative control outcomes likely to be affected by the same confounders as the outcome of interest, but that are unlikely to be directly affected by the exposure. These may be: (i) outcomes for which we believe there can be no plausible effect of the exposure; or (ii) records indicating whether an outcome of interest occurred before the patient was exposed to the treatment of interest (see Figure 3). If the instrumental variable is associated with a negative control outcome, then this suggests that there may be residual confounding and that assumption B of the instrumental variable analysis has been violated. The association of the instrument and the negative control outcome can be tested using linear regression. It is important to choose a negative control outcome that is affected by the same confounders as the outcome of interest, and which has sufficient variation to have adequate power.^23^ If a rare negative control outcome is used, then plots comparing the conventional linear and instrumental variable regression estimates would have wide confidence intervals and are likely to be uninformative.^24^

**Figure 4:**
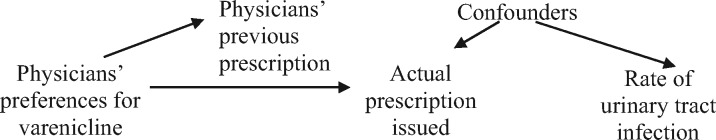
Using urinary tract infections as a negative control outcome to investigate the effects of prescribing varenicline.

The negative control outcome here can be a different diagnosis from the outcome of interest which occurs after prescription but is unlikely to be affected by treatment: an example in the case of varenicline is a urinary tract infection (see Figure 4). This is likely to be a suitable negative control outcome because a smoking cessation drug prescription is unlikely to be affected by a patient’s risk of developing a urinary tract infection. However, patients prescribed varenicline (as we will see) are generally healthier than those prescribed nicotine replacement therapy. Therefore they are likely to have a lower risk of urinary tract infection prior to prescription. Thus, urinary tract infections are affected by the same confounders, but are unlikely to be caused by varenicline. An example of a negative control outcome that is affected by treatment but occurred before the patient was prescribed treatment, could be a diagnosis of schizophrenia in the 6 months before the first smoking cessation prescription.

### Negative control populations

Another possible method to evaluate the instrumental variable assumptions is to use a negative control population. A negative control population has a similar confounding structure as the population of interest but was not exposed to the treatment of interest. In the context of physician prescribing preferences, the negative control population comprises patients that consulted with a GP who recently prescribed the medication of interest to another patient, but the negative control patient attended the GP for an unrelated reason and were not themselves prescribed the medications of interest (see Figure 3). In the case of smoking cessation therapies, these patients may not themselves be smokers. If the GP’s preferences have no direct effect on their patients’ outcomes then the instrumental variable, the physicians’ previous prescription, should not be associated with the outcomes in the negative control population. This is because a GP’s preferences for smoking cessation medications cannot directly affect the outcomes of patients who were not prescribed smoking cessation medications. If the proposed instrument is associated with any outcomes in the negative control population, this suggests that it may be operating through another mechanism.

### Bias component plots

Historically, studies using instrumental variables have reported tables of covariate balance across the exposure and the proposed instrument.^25^ Under the assumption that the structure of the observed confounding is similar to the unobserved confounding, we can potentially make inferences about the relative bias of the conventional linear and instrumental variable regression due to residual confounding. The confounders of the exposure-outcome relationship are not necessarily the same as the confounders of the instrument-outcome relationship. There is a substantial literature that describes methods to investigate the relative bias due to observed confounders. Brookhart and Schneeweiss (2007) described how to use the ‘prevalence difference ratio’ to investigate the relative bias.^26^ This is the ratio of the difference in an observed dichotomous confounder across values of the exposure and values of the instrument. However, this statistic does not directly account for the strength of the instruments. Brookhart and Schneeweiss conclude that if the prevalence difference ratio is smaller than the strength of the instrument, then the instrumental variable results are likely to have a lower asymptotic bias. Baiocchi and colleagues (2014) recommend generating a single statistic by dividing the prevalence difference ratio by the strength of the instrument to calculate what they term the ‘bias ratio’.^24^ Jackson and Swanson (2015) illustrated how simple plots of the associations of instrument and exposures with observed confounders can be misleading about the relative bias of instrumental variable and conventional linear regression.^27^ These methodological papers agree that one can only compare the relative bias of the two approaches if the fact that the instrument only explains a small proportion of the variation in the exposure is accounted for.^24^^,^^26^^,^^27^ To see why, compare the following expression for bias of the linear regression if the covariate *C* is omitted:

$$bias\left(OLS\right)/\beta_{C}=E\left[C|X=1\right]-E\left[C|X=0\right]$$.

where $\beta_{C}$ is the direct effect of C on the outcome. The bias in the Wald estimator if covariate *C* is omitted is:

$$bias(IV)/\beta_{C}=\frac{E\left[C|Z=1\right]-E\left[C|Z=0\right]}{E\left[X|Z=1\right]-E\left[X|Z=0\right]}$$

For comparisons, we normalize $\beta_{C}=1$. Jackson and Swanson argue that these estimated biases should be presented graphically using bias plots to aid interpretation.^27^

### Bias components without confidence intervals are uninformative

One limitation of these methods is that they ignore sampling variability, so the calculated differences could simply be due to chance. Furthermore, sampling variability will have a larger impact on the instrumental variable results because the instrumental variable estimates are less precise. Therefore, sampling variability must be taken into account when assessing bias. The simplest way to do this is to present confidence intervals around both the treatment and instrumental variable biases components and present a statistical test for differences between the terms.

Under the assumption of a constant effect of treatment, we can test whether the linear regression or instrumental variable bias component is bigger using a modified Hausman test. This test can be estimated using generalized method of moments; see online code repository for statistical code for this test at [https://github.com/nmdavies/varenicline-cprd-neg-control/]:

$$\frac{\left({\hat{\beta }}_{iv}-{\hat{\beta }}_{ols}\right)}{sqrt\left(v\hat{a}r\left({\hat{\beta }}_{iv}-{\hat{\beta }}_{ols}\right)\right)}∼N(0,1)$$

where ${\hat{\beta }}_{ols}\;$and ${\hat{\beta }}_{iv}$ are the ordinary least squares regression and instrumental variable regression estimates of the bias component terms. The null hypothesis of this test is that there is no difference between the linear regression and instrumental variable bias components. The alternative hypothesis is that there are differences. If there is little evidence of systematic differences between the instrumental variable and linear regression bias components, then we cannot say with any certainty which is bigger and it is difficult to draw any strong conclusions about the likely relative bias of the conventional linear and instrumental variable estimators. This is because any differences in the bias components could just be due to sampling variability, not differences in the true underlying distributions in the population or the true underlying distribution of unobserved confounders. Covariates which have systematic differences between the conventional linear and instrumental variable regression bias components are informative about the relative bias. We can illustrate this point using a simple simulation of a hypothetical analysis. Consider the following data-generating process:

v,w,u∼N(0,1)

The proposed instrument is distributed as an independent dichotomous variable. Therefore the exclusion restriction is valid:

z∼bernoulli(0.2)

Without loss of generality, assume that we have 10 potential (but not true) dichotomous confounders, j=1,. .,10:

$$c_{j}∼bernoulli\left(0.2\right)$$

Let the dichotomous exposure equal:

x=1(zγ+ u+w>d),

where 1(a)=1 if a and 0 otherwise, and γ is the strength of the effect of the instrument on the exposure, we set γ=0.5. We set the parameter d to ensure that Pr⁡[x=1]=0.2. The outcome is a continuous variable equal to:

y=xβ+u+v

**Figure 5:**
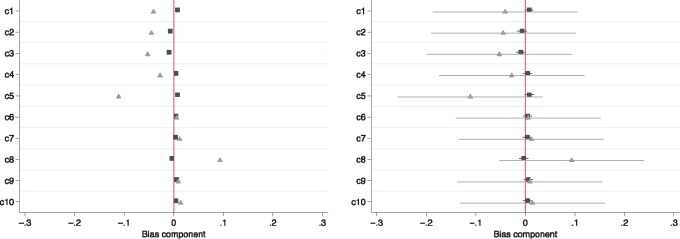
Bias component plots (left), are not informative without confidence intervals (right). Simulated bias component terms for 10 potential confounders (indicated c1 to c10) for the actual prescription (▪) and proposed instrument (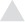). Simulation of 10 potential confounders when the instrument is valid. Using bias component plots alone we would erroneously conclude that the instrumental variable bias components were systematically larger than the linear regression bias components. Once we add confidence intervals to the point estimates, it becomes clear that the differences in components are entirely consistent with chance. There is no evidence from these potential confounders that the linear and instrumental variable regression bias component differ.

Conventional linear regression will suffer from bias due to the confounder u. We set the effect of the exposure, β=0.5, and N=10,000. The left panel of Figure 5 presents bias components without confidence intervals as recommended by Jackson and Swanson (2015).2^7^ From this figure, we would erroneously conclude that the instrumental variable analysis has larger bias components than conventional regression,n as the instrumental variable bias components are larger. However, in this simulated example, we know for certain that the instrumental variable analysis is asymptotically unbiased. The right panel of Figure 5 adds confidence intervals around the point estimates. The confidence intervals make it clear that there are no systematic and detectable differences in the bias components. Therefore bias component plots are not interpretable without confidence intervals.

### Selecting on (non-) treatment

Swanson and colleagues have suggested, using a simulated data-generating process, that instrumental variable studies can suffer from collider bias if analyses are restricted to patients who received a specific set of treatments, or if untreated patients are excluded.^29^ An example might be a study of smoking cessation treatment that ignored all smokers who chose not to take any medication. At present, it is not clear how pervasive this bias is in empirical pharmacoepidemiological studies. We can use the simulation described by Swanson and colleagues to investigate whether this bias is likely to be detectable using the methods described above. We modified their simulation to have a proxy (measured) confounder which had only a weak correlation with the true confounder (r^2 ^= 0.01) and found that if we restricted the analysis to treated patients, the instrumental variable bias component was detectable and an order of magnitude larger than the linear regression bias component. Therefore, whereas this bias is possible in empirical pharmacoepidemiological studies, it is likely to be detected by the statistics described above. The full statistical code of this analysis is available online at [https://github.com/nmdavies/varenicline-cprd-neg-control/].

## Application of negative controls and bias plots

### Study design and population

We illustrate the use of negative controls and bias plots using a sample from the Clinical Practice Research Datalink (CPRD) in which we investigated the effects of varenicline on suicide and self-harm, and depression.^18^ We were concerned that multivariable adjusted estimates of the effect of varenicline would suffer from residual confounding due to healthy user bias. Patients prescribed varenicline were healthier in almost all ways we could measure, and they were potentially healthier in ways we could not measure as well. This means we may underestimate the effect of varenicline on adverse outcomes. We used conventional multivariable adjusted regression and an instrumental variable analysis using physicians’ prescribing preferences for varenicline versus nicotine replacement therapy as an instrument.^18^ A description of the study cohort characteristics is presented in Table 1. Physicians who prescribed varenicline to their previous patient were 24 percentage points [95% confidence interval (CI): 23, 25] more likely to prescribe varenicline to their subsequent patients than physicians who previously prescribed nicotine replacement therapy (partial F-statistic = 1011.5). The large value of the partial F-statistic found here indicates that the instrument is strongly associated with the exposure.

**Table 1:** Description of baseline confounders of patients prescribed varenicline or nicotine replacement products

	Varenicline		Nicotine replacement products	
	*N* = 52981		*N* = 122159	
	(%)	SD	(%)	SD
Male	49.3		45.9	
Age (years)^a^	44.3	13.1	46.1	15.6
Prescribed in 2007	12.7		25.4	
Prescribed in 2008	19.4		17.7	
Prescribed in 2009	19.1		17.3	
Prescribed in 2010	20.1		14.7	
Prescribed in 2011	18.4		12.5	
Prescribed in 2012	10.3		7.1	
Number of GP visits in previous year^a^	6.3	8.9	12.0	11.2
Diagnoses in the previous year				
Autism	0.0		0.0	
Bipolar	0.0		0.2	
Current smoker	61.4		61.6	
Dementia	0.0		0.1	
Depression	3.8		6.5	
Eating disorder	0.0		0.1	
Hyperkinetic disorder	0.0		0.0	
Learning disability	0.0		0.1	
Neurotic disorder	2.0		3.4	
Other behavioural disorder	0.0		0.0	
Personality disorder	0.0		0.1	
Schizophrenia	0.0		0.3	
Alcohol misuse	0.9		1.7	
Probable self-harm	0.0		0.0	
Drug misuse	0.1		0.3	
Fractures	1.3		1.8	
Any psychiatric illness	6.0		10.5	
Chronic disease	7.7		11.2	
Prescriptions in the previous year				
Antidepressant	17.0		26.5	
Antipsychotic	2.9		6.4	
CNS stimulant	0.0		0.1	
Dementia medication	0.0		0.0	
Hypnotic anxiolytic	4.7		7.0	
Lithium	0.1		0.4	

This sample was larger than used Thomas and colleagues (2013) as in this study we also included patients who attended general practices that were not linked to the Hospital Episodes Statistics data.^18^

^a^Continuous variables, mean and standard deviation (SD) reported.

**Figure 6:**
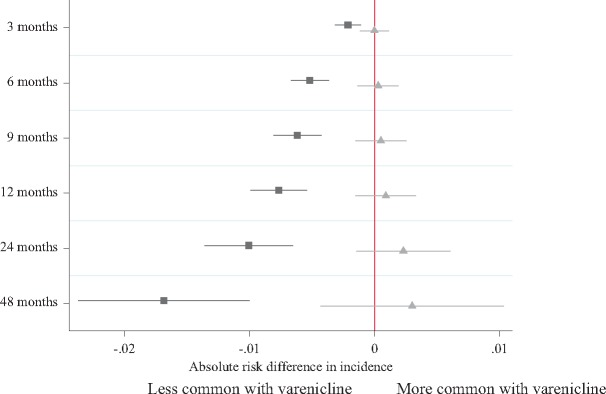
Negative control outcome: difference in the incidence of urinary tract infections in the four years after smoking cessation treatment for the index patients by actual prescription (▪) and the proposed instrument (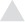). Horizontal lines indicate robust confidence intervals for each prescription. There is little evidence of differences in the prescribing history when the confidence intervals span zero on the axis.

We investigated whether varenicline was associated with a negative control outcome, urinary tract infections, as smoking cessation treatment is unlikely to affect the incidence of urinary tract infections. The conventional regression analysis suggests that patients prescribed varenicline were less likely to be subsequently diagnosed with a urinary tract infection (Figure 6). However, the instrumental variable analyses provided little evidence that varenicline caused urinary tract infections (Figure 6). The simplest explanation of these results is that the conventional regression analysis suffers from residual confounding, and the instrumental variable results do not (i.e. our instrument is not associated with potential confounders). We also investigated whether physicians’ preferences had any effects in a negative control population–individuals prescribed an antidepressant who consulted with a physician on the same day that the GP issued a smoking cessation medication to another patient. We found little evidence that the proposed instrument was associated with a range of outcomes in this population (Table 2). As there is little evidence that physicians’ preferences for prescribing varenicline directly affected their patients’ outcomes, this provides reassurance that they are potentially valid instruments (i.e. unconfounded).

**Table 2:** Association of proposed instrument and outcomes of other patients who saw the GP on the same day as they issued a smoking cessation therapy to an index patient (n = 101861)

	Robust linear regression		
	Risk difference*100	95% confidence interval	
		Lower	Upper
Male	0.77	0.02	1.52
Age (years)^a^	−0.35	−0.65	−0.05
Number of GP visits in previous year^a^	−0.35	−0.61	−0.08
Diagnoses in the previous year			
Autism	0.00	−0.02	0.02
Bipolar	−0.01	−0.05	0.03
Current smoker	0.28	−0.51	1.07
Dementia	0.00	−0.12	0.13
Depression	−0.04	−0.42	0.35
Eating disorder	−0.02	−0.05	0.02
Hyperkinetic disorder	0.00	−0.01	0.01
Learning disability	−0.01	−0.06	0.05
Neurotic disorder	0.56	0.29	0.84
Other behavioural disorder	−0.01	−0.04	0.01
Personality disorder	0.02	−0.03	0.07
Schizophrenia	0.00	−0.05	0.05
Alcohol misuse	0.08	−0.06	0.22
Probable self-harm	−0.01	−0.01	0.00
Drug misuse	0.03	−0.04	0.11
Fractures	0.03	−0.15	0.21
Any psychiatric illness	0.36	−0.10	0.81
Chronic disease	0.12	−0.34	0.59
Prescriptions in the previous year			
Antidepressant	0.44	−0.28	1.17
Antipsychotic	0.17	−0.18	0.52
CNS stimulant	0.02	−0.02	0.05
Dementia medication	0.00	−0.12	0.13
Hypnotic anxiolytic	−0.02	−0.40	0.35
Lithium	0.07	−0.01	0.16

Robust standard errors clustered by physician reported.

*Mean differences reported. Each outcome was defined as an event in the year after the index prescription.

**Figure 7:**
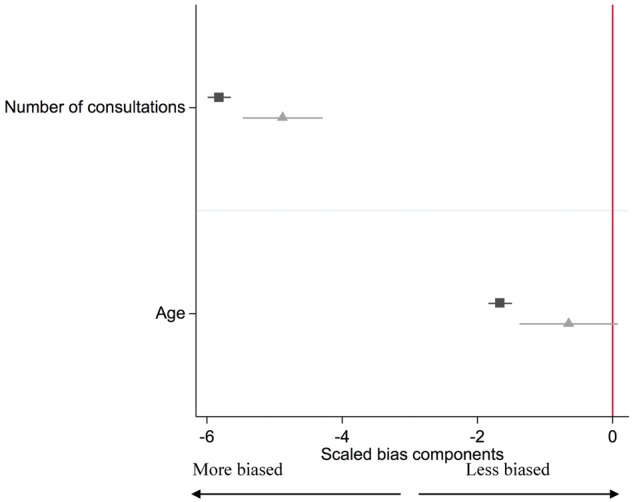
Bias component plots: difference in patient’s age and the number of consultations in the previous year by actual exposure (▪) and proposed instrument (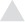). The figures for the instrumental variable results account for the strength of the instrument as described in Jackson and Swanson (2015).^27^ The horizontal lines indicate robust confidence intervals for each prescription. There is little evidence of differences in the prescribing history when the confidence intervals span zero on the axis.

**Figure 8:**
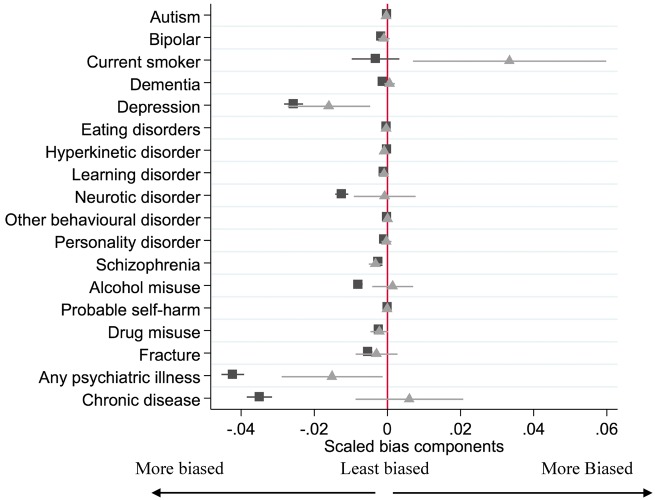
Bias component plots: difference in patients’ diagnoses in the previous year by actual exposure (▪) and proposed instrument (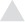). The figures for the instrumental variable results account for the strength of the instrument as described in Jackson and Swanson (2015).^27^ The horizontal lines indicate robust confidence intervals for each prescription. There is little evidence of differences in the prescribing history when the confidence intervals span zero on the axis.

**Figure 9:**
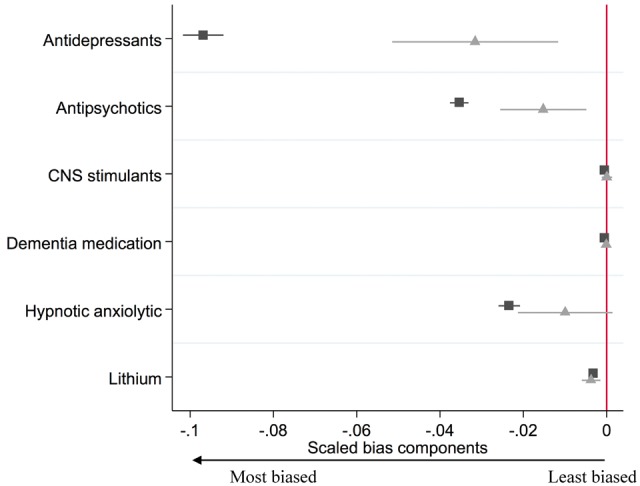
Bias component plots: difference in patients’ prescriptions received in the previous year by actual prescription (▪) and proposed instrument (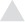). The figures for the instrumental variable results account for the strength of the instrument as described in Jackson and Swanson (2015).^27^ The horizontal lines indicate robust confidence intervals for each prescription. There is little evidence of differences in the prescribing history when the confidence intervals span zero on the axis.

The differences in baseline confounders for the actual exposure (prescription of varenicline rather than nicotine replacement therapy) and the proposed instrument (GP’s prescribing preference for varenicline versus nicotine replacement therapy) are shown in Table 3 and are presented in Figures 7–9. These results suggest that the instrumental variable (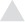) analysis would be less biased from these observed confounders than the conventional regression analysis (▪). This is because the instrumental variable bias terms are smaller than the conventional regression bias terms for number of consultations, age, diagnosis of a neurotic disorder, alcohol misuse, any psychiatric illness, chronic disease, prescription of antidepressants, antipsychotics and hypnotics.

**Table 3:** Estimates of the bias components for linear regression (equation 1) and instrumental variables (equation 2), and test for difference between the biases

	Linear regression bias component			Instrumental variable bias component			Test for difference^b^
	100*risk difference	Confidence interval		100*risk difference	Confidence interval		
N = 175,140		Lower	Upper		Lower	Upper	*P*-values
Male	3.06	2.50	3.63	1.05	−1.32	3.42	0.08
Age (years)*	−1.66	−1.84	−1.49	−0.65	−1.38	0.08	0.004
Number of GP visits in previous year*	−5.82	−5.99	−5.65	−4.88	−5.47	−4.29	5.84E-04
Diagnoses in the previous year								
Autism	−0.01	−0.02	0.00	−0.03	−0.07	0.01	0.47
Bipolar	−0.17	−0.20	−0.14	−0.09	−0.25	0.06	0.35
Current smoker	−0.33	−0.98	0.33	3.34	0.70	5.98	0.002
Dementia	−0.13	−0.16	−0.10	0.06	−0.08	0.20	0.01
Depression	−2.57	−2.83	−2.31	−1.60	−2.73	−0.47	0.07
Eating disorder	−0.03	−0.05	0.00	−0.03	−0.14	0.08	0.98
Hyperkinetic disorder	−0.02	−0.03	0.00	−0.09	−0.13	−0.04	2.11E-04
Learning disability	−0.11	−0.13	−0.08	−0.09	−0.22	0.04	0.79
Neurotic disorder	−1.25	−1.43	−1.07	−0.07	−0.92	0.77	0.004
Other behavioural disorder	−0.01	−0.03	0.00	0.00	−0.07	0.08	0.65
Personality disorder	−0.09	−0.12	−0.07	−0.03	−0.16	0.11	0.34
Schizophrenia	−0.25	−0.29	−0.22	−0.32	−0.51	−0.14	0.47
Alcohol misuse	−0.80	−0.92	−0.68	0.14	−0.41	0.70	6.64E-04
Probable self-harm	0.00	−0.01	0.00	0.00	−0.03	0.03	0.90
Drug misuse	−0.24	−0.29	−0.18	−0.22	−0.47	0.02	0.90
Fractures	−0.54	−0.67	−0.41	−0.30	−0.87	0.28	0.39
Any psychiatric illness	−4.23	−4.54	−3.92	−1.51	−2.89	−0.13	5.00E-05
Chronic disease	−3.50	−3.85	−3.16	0.60	−0.87	2.07	6.50E-09
Prescriptions in the previous year							
Antidepressant	−9.68	−10.17	−9.20	−3.15	−5.15	−1.16	1.06E-11
Antipsychotic	−3.54	−3.76	−3.32	−1.52	−2.55	−0.49	7.78E-05
CNS stimulant	−0.05	−0.08	−0.02	0.01	−0.12	0.13	0.39
Dementia medication	−0.04	−0.06	−0.03	−0.01	−0.09	0.07	0.37
Hypnotic anxiolytic	−2.34	−2.60	−2.08	−0.99	−2.13	0.14	0.01
Lithium	−0.32	−0.37	−0.28	−0.37	−0.60	−0.15	0.64

*Mean differences reported. Robust standard errors allowing for general form heteroskedasticity clustered on physician.

^b^Test for differences between the conventional regression and instrumental variable regression bias is $\left({\hat{\beta }}_{iv}-{\hat{\beta }}_{ols}\right)/\sqrt{v\hat{a}r\left({\hat{\beta }}_{iv}-{\hat{\beta }}_{ols}\right).}$

There are some caveats to these approaches. First, we cannot directly measure all confounders so must be cautious in assuming that the approaches provide conclusive proof that the instrument is valid. Second, using these approaches for one exposure-outcome association may not generalize to the instruments for other treatments. Third, the bias component terms assume a constant treatment effect. This means it is unclear whether these bias terms provide valid inferences about the relative bias when there are binary outcomes or heterogeneous treatment effects.

## Conclusion

We have demonstrated how negative control populations, negative control outcomes and covariate balance tests, when appropriately applied, can be used to investigate the relative biases of instrumental variable analysis and conventional regression. These approaches could be useful to researchers for interpreting evidence from studies reporting and comparing conventional and instrumental variable analysis, and ultimately improve the strength of the evidence provided to clinicians and policy makers.

## Funding

This work was supported by the Medical Research Council [MR/N01006X/1], the National Institute for Health Research (NIHR) Health Technology Assessment (HTA) programme [project number 14/49/94]. The Integrative Epidemiology Unit is supported by the Medical Research Council and the University of Bristol [MC_UU_12013/6, MC_UU_12013/9]. A.E.T., M.R.M. and G.T. are members of the UK Centre for Tobacco and Alcohol Studies, a UKCRC Public Health Research: Centre of Excellence. Funding from the British Heart Foundation, Cancer Research UK, Economic and Social Research Council, Medical Research Council and the National Institute for Health Research, under the auspices of the UK Clinical Research Collaboration, is gratefully acknowledged. K.H.T. is funded by a Clinical Lectureship from the National Institute for Health Research. R.M.M. is supported by Cancer Research UK programme grant [C18281/A19169] (the Integrative Cancer Epidemiology Programme). No funding body has influenced data collection, analysis or its interpretations. The views and opinions expressed therein are those of the authors and do not necessarily reflect those of the HTA programme, NIHR, NHS or the Department of Health.

Glossary

- **Instrumental variable:** a variable associated with the treatment of interest, but independent of confounding factors and having no direct effect on the outcome.
- **Physicians’ prescribing preferences:** the physicians’ preferences for prescribing one medication over another. It is not normally possible to directly measure physicians’ preferences, so most studies use the prescription they issued to their previous patients as a proxy.
- **Negative control outcome:** an outcome which the researcher believes should not be affected by the exposure or the proposed instrumental variable.
- **Negative control population:** a population in which the researcher believes the exposure or instrumental variable will not affect or be related to the outcome.
- **Bias component plot: **a graph depicting the relative bias of conventional regression and instrumental variable regression using observed covariates.
- **Latent variable:** a variable in a statistical model which is unobserved.
- **Collider bias:** if a variable, ‘a collider’, is caused by both the exposure and the outcome and is conditioned or selected on, then the conditional exposure-outcome association will be biased. This bias is referred to as collider bias.
- **Confounding by indication:** indications for treatment, such as blood pressure or cholesterol level, affect the likelihood of treatment with specific medications and can also affect the likelihood of an outcome. Thus indications confound the observed association of the treatment and outcomes, and hence this association is likely to be a biased estimate of the causal effects of treatment.
- **Hausman test for endogeneity:** test for differences between the conventional regression and instrumental variable results.
- **Partial F-test:** test used to evaluate the strength of the association between the instrumental variable(s) and the exposure, analogous to sample size in a randomized trial.
